# Syphiliphobia

**Published:** 1851-07-01

**Authors:** 


### SYPHILIPHOBIA.

Mr. Acton, in his recently-published work on the Diseases of the Reproductive Organs, has done much service in directing professional attention to a class of affections hitherto almost overlooked by the practitioner. He says—

"This class of complaints stands in direct opposition to feigned diseases; instead of our patients simulating certain affections or complaining of sensations which they themselves well know are for the mere purpose of misleading the medical man, the syphiliphobist describes only what by an exaltation of nervous sensibility he fully believes he sees or feels. Like hysteria, syphiliphobia will assume every form of venereal disease found or described in books, and in a tenfold degree, or like hypochondriasis, every trifling ailment will be exaggerated till the medical man is unable to distinguish what his patient really feels and what he supposes he feels.

"Did isolated cases only now and then occur, perhaps they might not deserve attention, but so numerous are they in a large capital like London, so anxious are the sufferers to obtain relief by consulting every man who can be supposed to offer them any means of relief, that they spend fortunes in travelling about and visiting every quack or novel quidnunc who gulls the public by assuming a knowledge which he does not possess.

"I have been consulted by a great number of persons who are fearful they suffer from syphilis in one form or another; and although many of these sufferers can be said to have syphilis only in their imagination, others have presented anomalous symptoms of disease which might lead the best educated medical man to waver, or doubt if it really was syphilis he was called on to treat and not the phantom above spoken of. The mistakes are most liable to occur from the surgeon depending upon the history given by the patient, rather than by the appearance which he meets with." —pp. 602-3.

He again observes—

"Sometimes the patient accuses the bladder, at other times the prostate, as being the seat of very peculiar symptoms, which have only this in common,—that without any apparent cause or symptom, his sufferings are exaggerated to a degree that we do not really meet with in the disease the patient supposes himself affected with. A most lamentable case of this nature came before the public lately, in consequence of the sufferer having committed suicide.

"There is something very peculiar in the aspect of this class of patients, which, coupled with the exaggerations of symptoms, leaves the surgeon in little doubt on the nature of the complaint; but although the diagnosis may be easy, the treatment is by no means successful."—p. 607.

We have, in the course of our practice, seen several cases of the kind. This affection has occurred in persons of very sensitive and highly wrought minds, who have been guilty of some slight impropriety, and have been impressed with an idea of being affected with various forms of *lues*.

Mr. Acton remarks—

"The inmate of many a lunatic asylum could give us a sad catalogue of errors of diagnosis; did he possess all his reasoning faculties, he could tell us that the monomania syphilitica was countenanced early in life by many a designing knave, who robbed him of his money while encouraging his fancies; that this same charlatan, professional or extra limites, thought it necessary to carry out his views by frequent cauterization, which had terminated in the present affections of the genital organs, that virtually produced the disease he once so much dreaded. But, poor fellow! this view of the case is happily not present to his mind, and he goes to the grave the victim of his own imagination, and a martyr to the injudicious treatment which has been pursued."—p. 608.

In a future number we may revert to this important subject.

---

